# IgG N-Glycosylation Is Altered in Coronary Artery Disease

**DOI:** 10.3390/biom13020375

**Published:** 2023-02-16

**Authors:** Barbara Radovani, Frano Vučković, Aldo P. Maggioni, Ele Ferrannini, Gordan Lauc, Ivan Gudelj

**Affiliations:** 1 Department of Biotechnology, University of Rijeka, 51000 Rijeka, Croatia; 2 Genos Glycoscience Research Laboratory, 10000 Zagreb, Croatia; 3 Heart Care Foundation ANMCO Research Center, 50100 Florence, Italy; 4 CNR Institute of Clinical Physiology, 56100 Pisa, Italy; 5 Faculty of Pharmacy and Biochemistry, University of Zagreb, 10000 Zagreb, Croatia

**Keywords:** immunoglobulin G, N-glycans, glycosylation, inflammation, coronary artery disease, cardiovascular disease

## Abstract

Coronary artery disease (CAD) is the most common cardiovascular disease (CVD), and previous studies have shown a significant association between N-glycosylation, a highly regulated posttranslational modification, and the development of atherosclerotic plaques. Our aim was to determine whether the N-glycome of immunoglobulin G (IgG) is associated with CAD, as N-glycans are known to alter the effector functions of IgG, which may enhance the inflammatory response in CAD. Therefore, in this study, we isolated IgG from subjects with coronary atherosclerosis (CAD+) and from subjects with clean coronaries (CAD−). The purified IgGs were denatured and enzymatically deglycosylated, and the released and fluorescently labelled N-glycans were analysed by ultra-high performance liquid chromatography based on hydrophilic interactions with fluorescence detection (HILIC-UHPLC-FLR). Sex-stratified analysis of 316 CAD− and 156 CAD+ cases revealed differences in IgG N-glycome composition. The most notable differences were observed in women, where the presence of sialylated N-glycan structures was negatively associated with CAD. The obtained chromatograms provide insight into the IgG N-glycome composition in CAD as well as the biomarker potential of IgG N-glycans in CAD.

## 1. Introduction

Cardiovascular diseases (CVDs), a group of diseases affecting the heart and blood vessels, are the most common non-communicable diseases globally, responsible for an estimated 18.6 million deaths in 2019 [1]. Coronary artery disease (CAD) is the most prevalent cardiovascular disease, resulting from chronic inflammation of the coronary arteries due to the formation of atherosclerotic plaques [2], and its presence is a significant marker of adverse cardiovascular (CV) events. Most preventive measures for CAD are based on the traditional risk factors (hyperlipidemia, diabetes mellitus, smoking and hypertension) [3], yet people with a high-risk profile do not consistently develop CAD, whereas others classified as low or intermediate may develop CAD [4,5,6,7,8]. Therefore, the discovery of a new risk factor and the potential opportunity to influence it is extremely important.

Glycosylation is one of the most common and important posttranslational modifications of proteins. It involves a series of enzymatic reactions in the synthesis of carbohydrate structures (glycans) attached to the protein backbone [9]. Unlike protein synthesis, which is defined by the nucleotide sequence in the genome, glycosylation has no direct genetic template but is a consequence of the current state of the organism. The dynamic interaction of enzymes, transcription factors, ion channels, and other proteins results in considerable variability in the glycome [9]. Glycans on the surface of proteins play key roles in a variety of biological processes, including stability and regulation of protein function, intercellular interaction, intracellular signalling, and cellular immunogenicity [9]. Glycome composition and complexity greatly influence protein effector functions in regulating the inflammatory response and modulating innate and adaptive immunity [10]. Accordingly, N-glycome as a novel biological marker is becoming increasingly important due to its sensitivity to changes that occur in the organism during (patho)physiological conditions and diseases, including CVD [11,12,13].

A growing body of research shows that alterations in protein glycosylation are involved in the development of cardiovascular disease through various molecular mechanisms and thus have significant biomarker potential for disease development and progression as well as for therapeutic monitoring [13]. For example, in atherosclerosis, which often underlies CVD, atherosclerotic plaque formation and arterial lumen narrowing occur as a result of the inflammatory process of accumulation of leukocytes, lipoproteins, and other cells in the arterial intima. Transmigration of leukocytes is mediated by their interaction with highly glycosylated adhesion molecules on the endothelial surface [14]. Pro-inflammatory cytokines contribute to altered glycosylation of endothelial cells, leading to dysregulation of adhesion molecule function at sites of early atherosclerotic plaque development [15,16]. In addition, glycosylation of lipoproteins is increasingly proving to be critical to the pathophysiology of atherosclerosis [17]. It has been shown that certain monosaccharides can significantly affect the process of cholesterol accumulation and efflux, opening the possibility for the development of new therapeutic and diagnostic approaches for the treatment of the disease [18]. In addition to significant changes in the glycosylation of endothelial cells and lipoproteins, there is the systematic response of an organism to inflammation; an increase in plasma protein concentration in response to inflammation [19] is accompanied by significant changes in their glycome [20].

Immunoglobulin G (IgG) is the most abundant antibody in human blood and a very important effector molecule in the immune response. IgG Fab fragment (antigen-binding fragment) specifically recognizes and binds various antigens, while the Fc fragment (crystallizable fragment) performs the effector function by binding to various Fc receptors [21]. The IgG molecule contains a conserved N-glycosylation site at position asparagine (Asn) 297 on each heavy chain of the Fc fragment. Fc glycosylation of IgG stabilizes the structure of the Fc fragment and is critical for regulating the anti-inflammatory and pro-inflammatory effector functions of IgG. For instance, fully sialylated IgG N-glycans exhibit anti-inflammatory properties, whereas pro-inflammatory changes in the IgG N-glycome are associated with decreased sialylation and increased levels of agalactosylation and bisecting N-acetylglucosamine (GlcNAc) [22]. Given that the IgG N-glycosylation profile is altered in inflammation [22] and a variety of diseases [11], it is not unexpected that changes in IgG N-glycosylation have been recognized in CVDs [13]. Moreover, IgG N-glycans have been associated with the predominant pathology of CVD– atherosclerosis. Precisely, a higher abundance of bisecting GlcNAc was positively associated with the presence of atherosclerotic plaques in carotid and femoral arteries, whereas the absence of bisecting GlcNAc and an increase in sialylation were negatively associated [23]. Furthermore, altered glycosylation of IgG has been shown to be a risk factor for the development of CVD, independent of other known risk factors [23,24]. Lastly, a recent study described an IgG N-glycome-based index for assessing and predicting cardiovascular health [25]. Therefore, considering the significant role that glycosylation plays in the development of CVD, distinct IgG glycosylation traits could stratify risk phenotypes for CAD development.

Considering previous research and the importance of glycosylation in CVD and the fact that there is no prospective evidence of altered IgG N-glycosylation in CAD, in this study, we performed an IgG N-glycome analysis of CAPIRE participants with angiographically diagnosed coronary atherosclerosis (CAD+) and participants with clean coronaries (CAD−). Our primary objective was to investigate the cross-sectional association between IgG N-glycome composition and CAD, as well as differences in IgG N-glycosylation occurring during a two-year follow-up period after CAD diagnosis.

## 2. Materials and Methods

### 2.1. Study Population

CAPIRE (ClinicalTrials.gov Identifier: NCT02157662) is a multicentre, prospective, observational study aimed at identifying new mechanisms promoting or protecting against coronary atherothrombosis; in its longitudinal phase, subjects are being followed for eight years. Details of the study have been reported [26]. Briefly, the study enrolled both male and female subjects aged 45 to 75 years, without previous clinical manifestations of CAD, including acute myocardial infarction, unstable angina, chronic stable angina, previous percutaneous or surgical coronary revascularisation, and heart failure, who underwent a 64-slice coronary computed tomography angiography (CCTA) because of suspected CAD. The inclusion and exclusion criteria have been illustrated elsewhere [26]. On the basis of CCTA, participants were grouped into CAD− (clean coronaries) and CAD+ (coronary atherosclerosis extended to >5 of the 16 segments according to the AHA classification [27]).

The whole study population had a peripheral venous blood sample taken at the inclusion point and after two years of follow-up. The samples were treated to obtain 0.5 mL whole blood, plasma, and serum aliquots and stored in a freezer at −70 °C. Samples of each subject enrolled in the study were collected in a single dedicated biological bank (SATURNE-1; Mario Negri Institute of Pharmacological Research, Milan, Italy). Personnel unaware of participant characteristics measured all biomarkers in a central laboratory in a single batch.

### 2.2. Methods

#### 2.2.1. Isolation of IgG from Human Plasma

IgG was isolated from the plasma samples by affinity chromatography, as described previously [28]. In brief, IgG was isolated in a high-throughput manner, using 96-well protein G monolithic plates (BIA Separations, Ajdovščina, Slovenia). Prior to use, monolithic plate was washed with ultrapure water maintained at 18.2 MΩ (Purelab Ultra, VWR, PA, USA) and equilibrated with phosphate-buffered saline (PBS). Plasma samples diluted 7-fold with PBS were applied to the protein G plate and washed with PBS to remove unbound proteins. IgG was eluted with 100 mM formic acid (Merck, Darmstadt, Germany) and immediately neutralized with 1 M ammonium bicarbonate (Thermo Fisher Scientific, MA, USA) to maintain the stability of IgG. The IgG eluates were dried overnight and stored at −20 °C until the deglycosylation procedure.

#### 2.2.2. Deglycosylation, Labelling, and Purification of IgG N-Glycans

Deglycosylation of IgG was performed as previously described [28]. Isolated IgG samples were denatured by the addition of sodium dodecyl sulphate (SDS) and incubation at 65 °C. The excess of SDS was neutralized with Igepal-CA630 (Sigma-Aldrich, MO, USA), and the N-glycans were released from the backbone of IgG by the addition of PNGase F (Promega, WI, USA). The released IgG N-glycans were labelled with fluorescent dye 2-aminobenzamide (2-AB). Excess dye and other reagents were removed from the samples using solid phase extraction (SPE). For the SPE step, an Acroprepadv 1 mL 0.2 μm wwPTFE plate (Pall, NY, USA) was used as the stationary phase. The labelled N-glycans were eluted with ultrapure water and stored at −20 °C until use.

#### 2.2.3. HILIC-UHPLC-FLR Analysis of 2-AB Labelled IgG N-Glycans

Fluorescently labelled N-glycans were analysed by ultra-high performance liquid chromatography based on hydrophilic interactions with fluorescence detection (HILIC-UHPLC-FLR) on a Waters Acquity UPLC H-class instrument consisting of a quaternary solvent manager, sample manager, and an FLR fluorescence detector. The instrument was under the control of Empower 3 software, build 3471 (Waters, Milford, MA, USA). Samples were kept at 10 °C before injection, and the separation temperature was 60 °C. Chromatographic separation of glycan structures was performed on Waters UPLC Glycan bridged ethylene hybrid (BEH) amide chromatographic columns (130 Å, 1.7 μm BEH particles, 2.1 × 100 mm). Solvent A was 100 mM ammonium formate, pH 4.4, while solvent B was LC-MS grade acetonitrile (ACN). Separation was performed with a linear gradient of 25–38% solvent A at a flow rate of 0.40 mL/min in a 29-min analytical run. Excitation and emission wavelengths were set at 250 and 428 nm, respectively. Data processing was performed using an automatic processing method with a traditional integration algorithm, after which each chromatogram was manually corrected to maintain the same intervals of integration for all the samples. Each chromatogram was separated into 24 N-glycan peaks (Figure S1), and the number of glycans in each peak was expressed as a percentage of the total integrated area (see [28]). Nine derived traits were calculated from the ratios of directly measured N-glycan peaks (GP1-GP24), each of which combined the glycans with the same structural characteristics; G0—glycans without galactose, G1—glycans with one galactose, G2—glycans with two galactoses, S—percentage of all glycans with sialic acid, F—core fucosylated glycans, B—glycans with bisecting N-acetylglucosamine (GlcNAc), FBS1/(FS1+FBS1)—glycans with bisecting GlcNAc in all fucosylated monosialylated structures, FBS1/FS1—core-fucosylated monosialylated glycans with and without bisecting GlcNAc, and FGS/(F+FG+FGS)—sialylated glycans of all fucosylated structures without bisecting GlcNAc. The calculation was performed according to the formulas shown in Table S1.

#### 2.2.4. Statistical Analysis

To remove experimental variation from the measurements, we performed normalization and batch correction on the UHPLC glycan data. To make measurements across samples comparable, we performed normalization by total area was performed. Prior to the batch correction, normalized glycan measurements were log-transformed due to right-skewness of their distributions and the multiplicative nature of batch effects. Batch correction was performed on log-transformed measurements using the ComBat method (R package sva), where the technical source of variation (which sample was analyzed on which plate) was modelled as batch covariate. To correct measurements for experimental noise, we subtracted the estimated batch effects from log-transformed measurements.

Association analyses between CAD status and baseline glycomic measurements were performed using a regression model. Analyses included glycan measurement as dependent continuous variable, CAD status was included as independent variable, with age and sex included as additional covariates. Additionally, interaction analyses were performed using a regression model where baseline glycan measurement was included in model as dependent continuous variable, CAD status, and interaction between CAD status and sex (CAD:sex) were included as independent variables, with age and sex included as additional covariates. To gain insight into associations between IgG N-glycome and CAD separately in women and men, we performed sex-stratified analyses. For sex-stratified analyses of association between CAD status and baseline glycomic measurements, two regression models were used. In model 1, glycan measurements were included as dependent continuous variable; CAD status was included as independent variable, with age included as additional covariate. In model 2, glycan measurements were included as dependent continuous variable, and CAD status was included as independent variable, with age, BMI, smoking, and diabetes included as additional covariates. For sex-stratified two-time points analyses of samples through their observation period, two linear mixed-effects models (LMM) were implemented. In first LMM model, glycan measurements were included as dependent continuous variable, time, CAD status, age, and interaction between time and CAD (CAD:time) status were modelled as fixed effects, while individual sample ID was modeled as a random intercept. In second LMM model, glycan measurements were included as dependent continuous variable, time, CAD status, age, BMI, smoking, diabetes, BMI:time, smoking:time, diabetes:time, and CAD:time were modelled as a fixed effects, while individual sample ID was modeled as a random intercept. Prior to analyses, glycan variables were all transformed to standard Normal distribution (mean = 0, sd = 1) by inverse transformation of ranks to Normality (R package “GenABEL”, function rntransform). Using rank transformed variables in analyses makes estimated effects of different glycans in different cohorts comparable as transformed glycan variables have the same standardized variance. False discovery rate was controlled using Benjamini–Hochberg procedure (function p.adjust(method = “BH”)).

Data were analyzed and visualized using R programming language (version 4.0.2).

## 3. Results

IgG N-glycome composition was analysed in CAPIRE participants classified by CCTA into CAD− (clean coronaries, n = 316) and CAD+ (diffuse coronary atherosclerosis with or without coronary stenosis, n = 156), whose samples were collected at the inclusion point and after the two-year follow-up period. Descriptive information on the included participants at the inclusion point is presented in Table 1. Briefly, participants in the CAD+ category were more often male, older, and heavier than subjects in the CAD− category; most clinical and metabolic parameters differed between the two groups, as expected. At the two-year follow-up point, a total of 285 samples were collected—CAD+ (101) and CAD− (184). The composition of the total IgG N-glycome (combined Fc and Fab glycans) was determined by HILIC-UHPLC-FLR analysis of 2-AB labelled glycans. An analysis of differences between CAD+ and CAD− cases was performed using a regression model, with age (and BMI, smoking, and diabetes) included as additional covariates to minimize the impact of these risk factors on the results. We did not detect statistically significant differences in IgG N-glycome when both sexes were combined (Table S2). However, a recently published study revealed substantial sex differences in IgG N-glycan-CVD risk associations [24]. To see if there is an effect modification by sex on the relationship between several IgG N-glycosylation traits and CAD, we performed interaction analysis (Table S3). We detected a suggestive sex-dependent sialylation-CAD and agalactosylation-CAD interaction, as well as significant interaction with several individual IgG glycan traits (Table S3). Therefore, we focused our study on the associations between IgG N-glycans and CAD separately in women and men.

**Figure 1:**
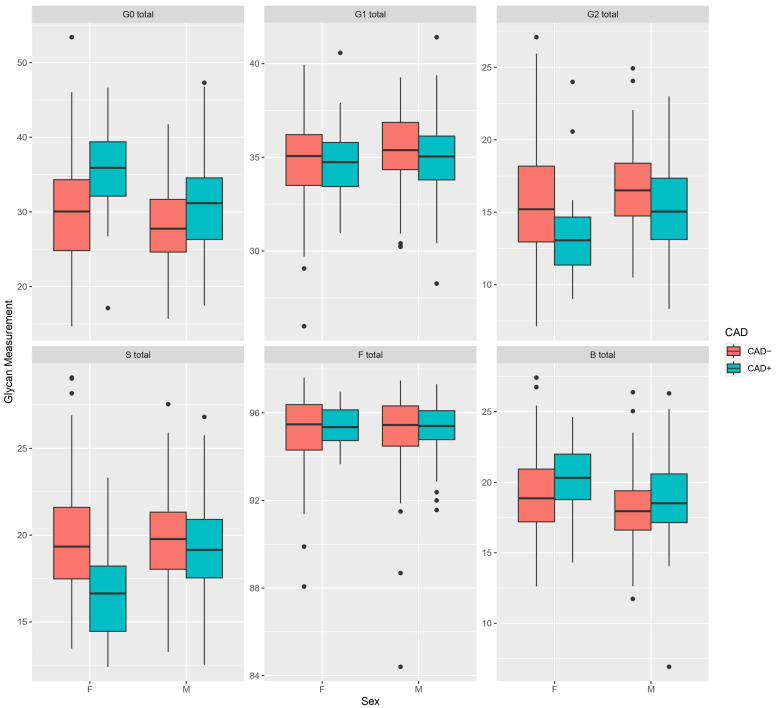
Sex-stratified relative abundance of main IgG glycome derived traits in CAD+ and CAD− cases at inclusion point. G0—agalactosylation, G1—monogalactosylation, G2—digalactosylation, S—sialylation, F—core fucosylation, B—bisecting GlcNAc, F—females, M—males, glycan measurement—expressed as percentage for each derived trait. Boxes represent the 25th and 75th percentiles. Lines inside the box represent the median.

To gain insight into the IgG N-glycome composition of participants at the time of inclusion in the study, we first compared the baseline glycomic measurements between the CAD+ and CAD− cases for both women and men. At the inclusion point, statistically significant differences were observed when comparing CAD+ and CAD− women, while the same was not observed in men. Based on model 1, in women, IgG sialylation was significantly negatively associated with CAD+ (Table 2, Figure 1). In particular, mono- and disialylated core-fucosylated biantennary glycans with or without bisecting GlcNAc (GP18, GP19, GP23, GP24) were significantly negatively associated with CAD+, while asialylated and agalactosylated N-glycan structure with a bisecting GlcNAc and core fucose (GP6) was positively associated with CAD+ (Table S4). In addition, agalactosylation was positively associated with CAD+; however, no significant associations were observed for mono- and digalactosylation (Table 2). In addition to the previously mentioned significant associations with individual glycan peaks, we observed nominal positive association between agalactosylated glycan structures (GP1, GP3, GP4) and CAD+, as well as a negative association with galactosylated glycan structures with terminal sialic acid (GP16, GP21). Interestingly, no differences were observed between CAD+ and CAD− women for total core fucosylation and bisecting GlcNAc, whereas sialylation of all core-fucosylated structures without bisecting GlcNAc was significantly negatively associated with CAD+ (Table 2). After further adjustment for BMI, diabetes, and smoking (Model 2), total sialylation and especially disialylated core-fucosylated glycan structures (GP23 and GP24) remained negatively associated with CAD+ (Table 2 and Table S4), but the association between agactosylation and CAD+ was only nominally significant (Table 2). In men, we did not observe any significant associations between IgG-derived traits and CAD+ (Table 2), only digalactosylated disialylated glycan structure with core fucose and bisecting GlcNAc (GP24) was positively associated with CAD (Table S4).

**Figure 2:**
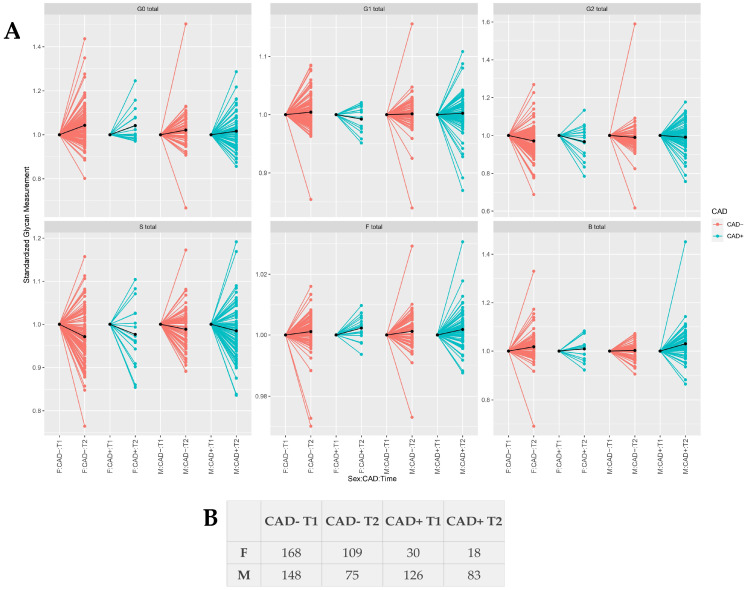
(**A**) Differences in sex-stratified IgG glycome composition during the follow-up period in CAD− and CAD+ cases. (**B**) Number of samples analyzed at each time point. G0—agalactosylation, G1—monogalactosylation, G2—digalactosylation, S—sialylation, F—core fucosylation, B—bisecting GlcNAc, F—females, M—males, T—timepoint. Median glycan values for each time point are bolded. Data is normalized to the first point.

Sequentially, we investigated whether the same trend of associations between IgG N-glycosylation and CAD continued during the two-year follow-up period after CAD diagnosis. Differences in IgG N-glycome composition observed during the follow-up period in CAD+ and CAD− cases are shown in Figure 2. Due to high variability, results after the two-year follow period showed only nominally statistically significant associations of IgG N-glycosylation profile with CAD+ participants (Table 3 and Table S5). Interestingly, none of the associations between IgG N-glycome and CAD+ women, observed at the inclusion point, remained significant after the two-year follow-up period. In men, a positive association between bisecting GlcNAc in all core-fucosylated monosialylated structures and CAD+ was observed, but statistical significance was lost after adjustment for multiple testing. In addition, a positive association between core-fucosylated galactosylated N-glycan structures with bisecting GlcNAc (GP10, GP13) and CAD+ was demonstrated in men (nominal *p*-value < 0.05, see Table S5). Consistent differences in the abundance of other derived traits, such as galactosylation, sialylation, and core fucosylation, were not observed between CAD+ and CAD− men.

## 4. Discussion

This study represents the first comprehensive analysis of IgG N-glycosylation in coronary artery disease (CAD). To investigate the association between coronary atherosclerosis and the composition of IgG N-glycome, we performed the N-glycomic analysis of IgG isolated from plasma of subjects with angiographically diagnosed coronary artery disease (CAD+) and subjects with clean coronary arteries (CAD−) collected at inclusion and after a two-year follow-up.

As previous studies demonstrated evidence of sexual dimorphism in CVD [29] as well as substantial sex differences of N-glycan-CVD risk associations [23,24,30], we performed sex-stratified IgG N-glycome analysis in CAD. In women with angiographically diagnosed CAD, the most extensive N-glycan differences were observed at the time of inclusion in the study, mainly revolving around sialylation. Sialylation was negatively associated with CAD after adjustment for traditional CVD risk factors such as age, BMI, diabetes, and smoking. Furthermore, the percentage of sialylation of all core-fucosylated structures without bisecting GlcNAc was significantly lower in CAD+ women when compared to CAD− women. Interestingly, this is consistent with what was observed by Menni et al. in their study [23]. They reported that sialylated glycans without a bisecting GlcNAc are negatively associated with the presence of subclinical atherosclerosis as well as cardiovascular risk. Along with sialylation, galactosylation also exhibited decreasing trend in CAD+ women. The loss of galactose in the IgG N-glycome has already been associated with higher pro-inflammatory IgG function by triggering complement activation via the alternative pathway and/or the lectin pathway after binding to mannose-binding lectin [22]. N-glycosylation is known to be affected by sex-specific modulation, and most differences in glycosylation have been attributed to sex hormones. Estrogen plays a direct role in modulating the galactosylation of IgG N-glycans [31]. Thus, galactosylation and sialylation of IgG have been reported to be higher in premenopausal women than in men, while an increase in agalactosylation has been associated with the transition to menopause [32]. Because the women participating in the study are of an age when they are in the transition to menopause [33], hormonal modulation cannot be excluded as a possible reason for a statistically nonsignificant difference between CAD+ and CAD− women after the two-year follow-up period.

In men, however, the differences were minor and revolved mainly around the presence of bisecting GlcNAc. At the inclusion point, GP24 was significantly positively associated with CAD. Furthermore, results after the two-year follow-up period demonstrated only a marginally positive association between core-fucosylated N-glycan structures with bisecting GlcNAc and CAD. However, this association was previously observed in a study that found sex-stratified associations between IgG N-glycans and the incidence of CVD [24]. Specifically, in men, agalactosylation and bisecting GlcNAc were positively associated, whereas digalactosylation and monosialylation were negatively associated with CVD risk. In addition, a study investigating the role of IgG N-glycome in subclinical atherosclerosis showed that a higher abundance of bisecting GlcNAc in IgG N-glycome was positively associated with the presence of atherosclerotic plaques in carotid and femoral arteries, whereas sialylated glycans lacking bisecting GlcNAc were negatively associated [23]. Interestingly, an increased abundance of bisecting GlcNAc in the N-glycome of IgG is often associated with increased FcγRIII binding and enhanced antibody-dependent cell cytotoxicity (ADCC), explaining a more pro-inflammatory profile of IgG [22].

However, even though in this study, we found an association between IgG N-glycome and CAD, we were not able to illuminate the mechanism behind those associations. Thus, it is known that in addition to direct effects on the immunomodulatory function of IgG, alterations in the glycosylation profile have also been associated with cardiovascular risk factors [13], which account for most of the CVD burden [34]. Traditional CVD risk factors (age, sex, BMI, smoking, cholesterol level, systolic blood pressure, and diabetes) have been shown to be under the influence of several glycosylation traits that together can further exacerbate disease development [13]. There are several different validated algorithms for assessing an individual’s risk for developing certain CVD events that use those traditional risk factors for the assessment [8,35,36,37,38]. One of these assessment tools is the 10-year atherosclerotic cardiovascular disease risk score (ASCVD), a single multivariable risk assessment tool used to estimate an individual’s 10-year CVD risk in a gender- and race-specific way [38]. In the study by Menni et al., they found that several IgG N-glycosylation traits were significantly associated with ASCVD risk score. More precisely, core-fucosylated N-glycans with bisecting GlcNAc were positively associated with higher ASCVD risk, whereas the opposite was observed for core-fucosylated sialylated N-glycans lacking bisecting GlcNAc, and, as mentioned previously, these same features were associated with subclinical atherosclerosis and those associations remained significant even after the adjustment for all risk factors that constitute the ASCVD risk score. Given the similarity of these observations to the results obtained from our study, this further supports the importance of the interplay of IgG N-glycans and the development of atherosclerotic CVDs.

This study, to our knowledge, is the first study to investigate the association of total IgG N-glycome with coronary artery disease. Nevertheless, there are limitations to our study. First, despite the multicenter design of the CAPIRE study, only European centers with Caucasian patients were involved. Therefore, our conclusions should not be extrapolated to non-European populations because the results may not reflect the general population of other countries. Second, patients might have changed their medication regimen or lifestyle after coronary CTA; we were not able to assess the impact of this possible bias on our results. Finally, our study lacks a validation cohort; however, we are planning to set up another study to validate our results.

In conclusion, our study demonstrated several significant cross-sectional associations between IgG N-glycosylation and CAD. Further research on IgG glycosylation as a potential biomarker for CVD prevention and early diagnosis is encouraged.

## Figures and Tables

**Table 1:** Characteristics of the participants at inclusion point.

	All Population (n = 472)	CAD− (n = 316)	CAD+ (n = 156)
Age (y), mean ± SD	59.9 ± 8.4	58.1 ± 8.3	63.7 ± 7.4
Women, %	42	53	19
BMI (kg/m^2^), mean ± SD	26 ± 4.2	26 ± 3.9	27.7 ± 4.5
Diabetes, %	12	7	21
Smoking, %	26	21	37

**Table 2:** Statistical analysis of IgG N-glycan derived traits in CAD+ versus CAD− cases at inclusion point.

Glycan Traits	Model 1 **								Model 2 ***							
	Women				Men				Women				Men			
	Effect	SE	*p*-Value	*p*_adj_-Value *	Effect	SE	*p*-Value	*p*_adj_-Value *	Effect	SE	*p*-Value	*p*_adj_-Value *	Effect	SE	*p*-Value	*p*_adj_-Value *
**S total**	−0.82	0.1705	**1.99 × 10^−6^**	**6.78 × 10^−5^**	0.03	0.1296	8.05 × 10^−1^	9.58 × 10^−1^	−0.63	0.1781	**3.89 × 10^−4^**	**2.65 × 10^−2^**	0.19	0.1306	1.40 × 10^−1^	4.92 × 10^−1^
**G0 total**	0.48	0.1599	**2.51 × 10^−3^**	**2.26 × 10^−2^**	0.1	0.1241	4.03 × 10^−1^	7.93 × 10^−1^	0.4	0.1708	**1.80 × 10^−2^**	1.53 × 10^−1^	−0.05	0.1249	6.81 × 10^−1^	9.38 × 10^−1^
**G1 total**	−0.03	0.204	8.76 × 10^−1^	9.58 × 10^−1^	−0.18	0.1293	1.56 × 10^−1^	4.25 × 10^−1^	−0.12	0.2207	5.65 × 10^−1^	9.11 × 10^−1^	−0.11	0.1335	3.90 × 10^−1^	8.21 × 10^−1^
**G2 total**	−0.28	0.1584	7.29 × 10^−2^	2.70 × 10^−1^	−0.17	0.1224	1.74 × 10^−1^	4.36 × 10^−1^	−0.26	0.1707	1.18 × 10^−1^	4.92 × 10^−1^	−0.05	0.1241	6.77 × 10^−1^	9.38 × 10^−1^
**F total**	−0.02	0.2032	9.38 × 10^−1^	9.80 × 10^−1^	0	0.1327	9.80 × 10^−1^	9.80 × 10^−1^	−0.01	0.2183	9.75 × 10^−1^	9.91 × 10^−1^	0.01	0.1386	9.54 × 10^−1^	9.91 × 10^−1^
**B total**	0.21	0.1915	2.58 × 10^−1^	5.65 × 10^−1^	0.19	0.1308	1.48 × 10^−1^	4.20 × 10^−1^	−0.06	0.1993	7.75 × 10^−1^	9.49 × 10^−1^	0.06	0.131	6.39 × 10^−1^	9.38 × 10^−1^
**FGS/(F + FG + FGS)**	−0.71	0.1702	**3.44 × 10^−5^**	**7.81 × 10^−4^**	0.01	0.1285	9.66 × 10^−1^	9.80 × 10^−1^	−0.56	0.18	**1.66 × 10^−3^**	**2.91 × 10^−2^**	0.13	0.1312	3.09 × 10^−1^	8.09 × 10^−1^
**FBS1/(FS1 + FBS1)**	−0.03	0.184	8.81 × 10^−1^	9.58 × 10^−1^	0.02	0.1301	8.86 × 10^−1^	9.58 × 10^−1^	−0.06	0.1988	7.73 × 10^−1^	9.49 × 10^−1^	0.00	0.136	9.91 × 10^−1^	9.91 × 10^−1^
**FBS1/FS1**	−0.03	0.184	8.81 × 10^−1^	9.58 × 10^−1^	0.02	0.1301	8.86 × 10^−1^	9.58 × 10^−1^	−0.06	0.1988	7.73 × 10^−1^	9.49 × 10^−1^	0.00	0.136	9.91 × 10^−1^	9.91 × 10^−1^

* False discovery rate was controlled using Benjamini–Hochberg method; ** Model 1—adjustment for age; *** Model 2—adjustment for age, BMI, diabetes, and smoking.

**Table 3:** Statistical analysis of IgG N-glycan derived traits in CAD+ versus CAD− cases during the two-year follow-up.

Glycan Traits	Model 1 **								Model 2 ***							
	Women				Men				Women				Men			
	Effect	SE	*p*-Value	*p*_adj_-Value *	Effect	SE	*p*-Value	*p*_adj_-Value *	Effect	SE	*p*-Value	*p*_adj_-Value *	Effect	SE	*p*-Value	*p*_adj_-Value *
**S total**	0	0.1025	9.98 × 10^−1^	9.98 × 10^−1^	−0.02	0.0623	7.49 × 10^−1^	9.41 × 10^−1^	0.06	0.109	5.99 × 10^−1^	9.54 × 10^−1^	−0.02	0.0667	7.18 × 10^−1^	9.90 × 10^−1^
**G0 total**	0.05	0.1027	6.09 × 10^−1^	9.41 × 10^−1^	−0.01	0.0648	8.26 × 10^−1^	9.62 × 10^−1^	0.01	0.1107	9.42 × 10^−1^	9.90 × 10^−1^	−0.02	0.068	7.84 × 10^−1^	9.90 × 10^−1^
**G1 total**	−0.16	0.1154	1.73 × 10^−1^	6.76 × 10^−1^	−0.01	0.0896	9.17 × 10^−1^	9.89 × 10^−1^	−0.1	0.1256	4.23 × 10^−1^	8.74 × 10^−1^	0.03	0.0925	7.75 × 10^−1^	9.90 × 10^−1^
**G2 total**	−0.03	0.1011	7.75 × 10^−1^	9.41 × 10^−1^	0	0.0621	9.79 × 10^−1^	9.98 × 10^−1^	−0.02	0.1098	8.42 × 10^−1^	9.90 × 10^−1^	0.02	0.0644	8.11 × 10^−1^	9.90 × 10^−1^
**F total**	0.1	0.1061	3.40 × 10^−1^	8.41 × 10^−1^	0.03	0.0811	6.74 × 10^−1^	9.41 × 10^−1^	0.15	0.1133	1.85 × 10^−1^	5.85 × 10^−1^	0.04	0.0838	6.09 × 10^−1^	9.54 × 10^−1^
**B total**	0.02	0.1092	8.73 × 10^−1^	9.62 × 10^−1^	0.16	0.0621	**8.96 × 10^−3^**	2.45 × 10^−1^	0.07	0.1204	5.83 × 10^−1^	9.54 × 10^−1^	0.2	0.0653	**2.41 × 10^−3^**	8.79 × 10^−2^
**FGS/(F + FG + FGS)**	0.05	0.1084	6.75 × 10^−1^	9.41 × 10^−1^	−0.03	0.0601	6.61 × 10^−1^	9.41 × 10^−1^	0.09	0.1182	4.56 × 10^−1^	8.74 × 10^−1^	−0.03	0.0635	6.13 × 10^−1^	9.54 × 10^−1^
**FBS1/(FS1 + FBS1)**	−0.08	0.1061	4.80 × 10^−1^	8.93 × 10^−1^	0.11	0.0566	6.24 × 10^−2^	6.09 × 10^−1^	−0.02	0.1163	8.60 × 10^−1^	9.90 × 10^−1^	0.12	0.0592	**4.66 × 10^−2^**	4.61 × 10^−1^
**FBS1/FS1**	−0.08	0.1061	4.80 × 10^−1^	8.93 × 10^−1^	0.11	0.0566	6.24 × 10^−2^	6.09 × 10^−1^	−0.02	0.1163	8.60 × 10^−1^	9.90 × 10^−1^	0.12	0.0592	**4.66 × 10^−2^**	4.61 × 10^−1^

* False discovery rate was controlled using Benjamini–Hochberg method; ** Model 1—adjustment for age; *** Model 2—adjustment for age, BMI, diabetes, and smoking.

## Data Availability

Not applicable.

## Supplementary Materials

The following supporting information can be downloaded at: https://www.mdpi.com/article/10.3390/biom13020375/s1, Table S1: IgG-derived N-glycan traits calculated out of 24 directly measured IgG N-glycan peaks; Table S2: Statistical analysis of associations between IgG glycosylation traits (24 directly measured and six derived) and coronary artery disease at the inclusion point and during the two-year follow-up period, after adjustment for age and sex; Table S3: Interaction analysis for IgG glycosylation traits (24 directly measured and 9 calculated derived) and CAD by sex; Table S4: Statistical analysis of sex-stratified associations between 24 quantitative IgG glycosylation traits and coronary artery disease at the inclusion point; Table S5: Statistical analysis of sex-stratified associations between 24 quantitative IgG glycosylation traits and coronary artery disease during the two-year follow up; Figure S1: Representative chromatogram of 2-AB labelled IgG N-glycans separated by HILIC–UHPLC. The integration areas, together with a major structure presented in each glycan peak, are given. Glycan peaks are numbered as used in the paper.
