# Supplementary material for: IgG N-Glycosylation Is Altered in Coronary Artery Disease
Source: Biomolecules. 2023 Feb 16;13(2):375. doi: 10.3390/biom13020375 (PMC9953309; doi:10.3390/biom13020375)
Supplement: Supplementary file 1 [file biomolecules-13-00375-s001.zip › biomolecules-2124726-supplementary.pdf]

Supplementary Materials

IgG N-glycosylation is altered in coronary artery disease

Barbara Radovani [1], Frano Vučković [2], Aldo P. Maggioni [3], Ele Ferrannini [4], Gordan Lauc [2,5] and Ivan Gudelj [1,2,*]

[1] Department of Biotechnology, University of Rijeka, Rijeka, Croatia; (B.R.) barbara.radovani@uniri.hr
[2] Genos Glycoscience Research Laboratory, Zagreb, Croatia; (F.V.) fvuckovic@genos.hr, (G.L.) glauc@genos.hr, (I.G.) igudelj@genos.hr
[3] Heart Care Foundation ANMCO Research Center, Florence, Italy; (A.P.M.) maggioni@anmco.it
[4] CNR Institute of Clinical Physiology, Pisa, Italy; (E.L.) ferranni@ifc.cnr.it
[5] Faculty of Pharmacy and Biochemistry, University of Zagreb, Zagreb, Croatia
* Correspondence: ivan.gudelj@uniri.hr

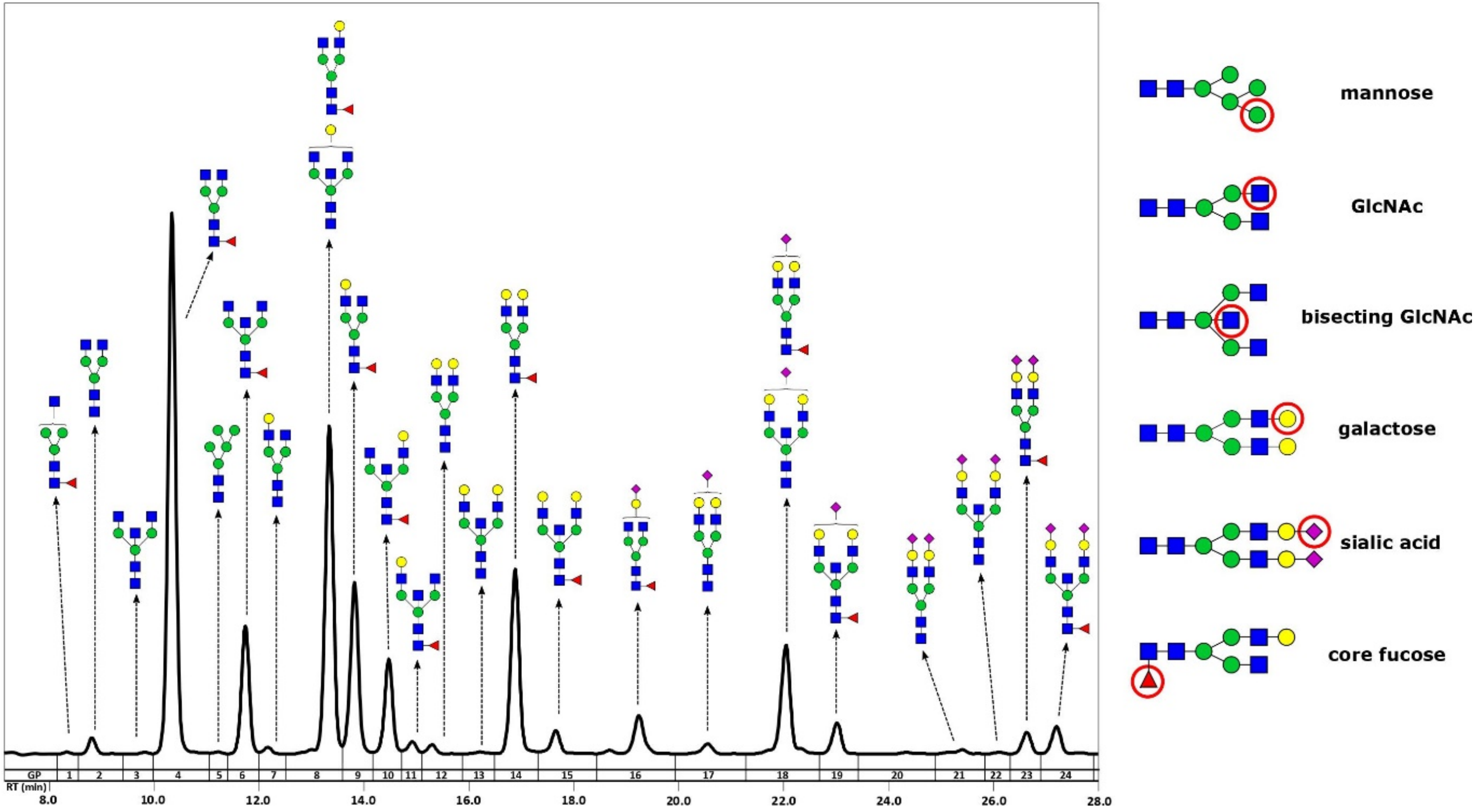

Figure S1. Representative chromatogram of 2-AB labelled IgG N-glycans separated by HILIC–UHPLC-FLR. The integration areas, together with a major structure presented in each glycan peak are given. Glycan peaks are numbered as used in the paper

Table S1. IgG derived N-glycan traits calculated out of 24 directly measured IgG N-glycan peaks

**Derived trait**	**Formula**
**Agalactosylation G0**	SUM(GP1 + GP2 + GP3 + GP4 + GP6) / GP_{total} * 100
**Monogalactosylation G1**	SUM(GP7 + GP8 + GP9 + GP10 + GP11) / GP_{total} * 100
**Digalactosylation G2**	SUM(GP12 + GP13 + GP14 + GP15) / GP_{total} * 100
**Sialylation S**	SUM(GP16 + GP17 + GP18 + GP19 + GP20 + GP21 + GP22 + GP23 + GP24) / GP_{total} * 100
**Core fucosylation CF**	SUM(GP1 + GP4 + GP6 + GP8 + GP9 + GP10 + GP11 + GP14 + GP15 + GP16 + GP18 + GP19 + GP23 + GP24) / GP_{total} * 100
**Bisecting GlcNAc B**	SUM(GP3 + GP6 + GP10 + GP11 + GP13 + GP15 + GP19 + GP22 + GP24) / GP_{total} * 100
**Bisecting GlcNAc in all fucosylated monosialylated structures FBS1/(FS1+FBS1)**	GP19 / SUM(GP16 + GP18 + GP19)
**Core fucosylated monosialylated with and without bisecting GlcNAc FBS1/FS1**	GP19 / SUM(GP16 + GP18)
**Sialylated glycans of all fucosylated structures without bisecting GlcNAc FGS/(F+FG+FGS)**	SUM(GP16 + GP18 +GP23) / SUM(GP16 + GP18 + GP23 + GP4 + GP8 + GP9 + GP14) * 100

Table S2. Statistical analysis of associations between IgG glycosylation traits (24 directly measured and six derived) and coronary artery disease at the inclusion point and during the two-year follow up period, after adjustment for age and sex

	Inclusion point				Two-year follow up point			
**Glycan trait**	**Effect**	**SE**	**p-value**	**p_{adj}-value***	**Effect**	**SE**	**p-value**	**p_{adj}-value***
**GP1**	0	0.1035	9.92E-01	9.92E-01	0.02	0.0597	7.74E-01	8.60E-01
**GP2**	0.06	0.1094	5.67E-01	7.09E-01	-0.1	0.0523	6.52E-02	4.89E-01
**GP3**	0.11	0.101	2.69E-01	5.04E-01	-0.01	0.0724	8.99E-01	8.99E-01
**GP4**	0.12	0.0988	2.27E-01	4.55E-01	-0.05	0.0484	2.76E-01	7.94E-01
**GP5**	-0.1	0.1073	3.29E-01	5.48E-01	-0.14	0.0969	1.45E-01	7.26E-01
**GP6**	0.21	0.1011	**3.99E-02**	4.25E-01	0.03	0.0409	4.56E-01	7.94E-01
**GP7**	0.01	0.1099	9.47E-01	9.88E-01	-0.05	0.0492	3.18E-01	7.94E-01
**GP8**	-0.11	0.1082	3.05E-01	5.37E-01	-0.03	0.0559	5.78E-01	7.94E-01
**GP9**	-0.19	0.108	8.04E-02	4.25E-01	-0.11	0.05	**2.39E-02**	3.59E-01
**GP10**	0.14	0.1095	1.88E-01	4.33E-01	0.11	0.0474	**1.83E-02**	3.59E-01
**GP11**	0.08	0.1082	4.78E-01	7.09E-01	0.02	0.0416	6.55E-01	7.94E-01
**GP12**	0.03	0.1065	7.83E-01	9.40E-01	-0.02	0.0494	6.62E-01	7.94E-01
**GP13**	-0.17	0.1062	1.03E-01	4.25E-01	0.07	0.0559	2.33E-01	7.94E-01
**GP14**	-0.14	0.0955	1.39E-01	4.25E-01	0.04	0.0489	4.67E-01	7.94E-01
**GP15**	-0.06	0.1061	5.66E-01	7.09E-01	0.09	0.0538	1.01E-01	6.04E-01
**GP16**	-0.09	0.1089	4.16E-01	6.56E-01	-0.01	0.0425	8.13E-01	8.71E-01
**GP17**	-0.02	0.1096	8.56E-01	9.51E-01	-0.07	0.0723	3.20E-01	7.94E-01
**GP18**	-0.15	0.1003	1.35E-01	4.25E-01	0.03	0.0497	5.73E-01	7.94E-01
**GP19**	-0.14	0.1091	2.08E-01	4.45E-01	0.02	0.05	7.07E-01	8.16E-01
**GP20**	-0.14	0.1073	1.82E-01	4.33E-01	-0.09	0.1096	4.12E-01	7.94E-01
**GP21**	-0.07	0.1089	5.12E-01	7.09E-01	0.02	0.1181	8.75E-01	8.99E-01
**GP22**	0.06	0.1096	5.60E-01	7.09E-01	0.06	0.0683	4.16E-01	7.94E-01
**GP23**	-0.25	0.1064	**1.98E-02**	4.25E-01	0.02	0.0534	6.57E-01	7.94E-01
**GP24**	-0.01	0.1092	9.55E-01	9.88E-01	0.04	0.0612	5.59E-01	7.94E-01
**G0 total**	0.15	0.0974	1.16E-01	4.25E-01	-0.04	0.0494	3.94E-01	7.94E-01
**G1 total**	-0.14	0.1085	1.81E-01	4.33E-01	-0.03	0.0637	5.89E-01	7.94E-01

**G2 total**	-0.14	0.0955	1.41E-01	4.25E-01	0.04	0.0492	3.98E-01	7.94E-01
**S total**	-0.18	0.104	8.91E-02	4.25E-01	0.03	0.0486	5.37E-01	7.94E-01
**F total**	-0.02	0.1093	8.25E-01	9.51E-01	0.06	0.0566	2.74E-01	7.94E-01
**B total**	0.15	0.1057	1.42E-01	4.25E-01	0.1	0.0487	**4.67E-02**	4.67E-01

*False discovery rate was controlled using Benjamini–Hochberg method

Table S3. Interaction analysis for IgG glycosylation traits (24 directly measured and 9 calculated derived) and CAD by sex

Glycan trait	p-value	p_{adj}-value*
**GP1**	**3.75E-02**	9.10E-02
**GP2**	**4.85E-02**	1.05E-01
**GP3**	**1.07E-02**	**3.51E-02**
**GP4**	**1.24E-02**	**3.51E-02**
**GP5**	6.93E-01	7.85E-01
**GP6**	**1.20E-02**	**3.51E-02**
**GP7**	6.40E-01	7.50E-01
**GP8**	9.35E-01	9.35E-01
**GP9**	7.84E-01	8.58E-01
**GP10**	2.55E-01	4.13E-01
**GP11**	2.16E-01	3.67E-01
**GP12**	5.79E-01	7.29E-01
**GP13**	6.23E-01	7.50E-01
**GP14**	5.25E-02	1.05E-01
**GP15**	2.76E-01	4.26E-01
**GP16**	**1.02E-02**	**3.51E-02**
**GP17**	**1.19E-02**	**3.51E-02**
**GP18**	**4.58E-04**	**3.11E-03**
**GP19**	**1.90E-02**	**4.96E-02**
**GP20**	1.81E-01	3.24E-01
**GP21**	**6.73E-03**	**3.27E-02**
**GP22**	1.80E-01	3.24E-01
**GP23**	**2.87E-04**	**2.44E-03**
**GP24**	**2.51E-05**	**3.98E-04**
**S total**	**3.81E-06**	**1.29E-04**
**B total**	3.78E-01	5.58E-01

**F total**	8.07E-01	8.58E-01
**G0 total**	**3.31E-03**	**1.87E-02**
**G1 total**	4.25E-01	6.02E-01
**G2 total**	5.00E-02	1.05E-01

*False discovery rate was controlled using Benjamini–Hochberg method

Table S4. Statistical analysis of sex-stratified associations between 24 quantitative IgG glycosylation traits and coronary artery disease at the inclusion point

	Model 1**								Model 2***							
	Women				Men				Women				Men			
**Glycan trait**	**Effect**	**SE**	**p-value**	**p_{adj}-value***	**Effect**	**SE**	**p-value**	**p_{adj}-value***	**Effect**	**SE**	**p-value**	**p_{adj}-value***	**Effect**	**SE**	**p-value**	**p_{adj}-value***
**GP1**	0.29	0.1873	**1.16E-01**	3.74E-01	-0.07	0.1267	5.95E-01	9.02E-01	0.33	0.2029	9.41E-02	4.92E-01	-0.09	0.1318	4.86E-01	8.83E-01
**GP2**	0.31	0.1966	1.15E-01	3.74E-01	0.01	0.133	9.40E-01	9.80E-01	0.22	0.2124	2.91E-01	8.09E-01	-0.08	0.1378	5.76E-01	9.11E-01
**GP3**	0.38	0.1699	**2.29E-02**	1.20E-01	0.08	0.1273	5.47E-01	9.02E-01	0.38	0.1811	**3.56E-02**	2.30E-01	-0.03	0.1307	8.26E-01	9.52E-01
**GP4**	0.39	0.167	**1.83E-02**	1.03E-01	0.06	0.1251	6.14E-01	9.02E-01	0.37	0.1784	**3.72E-02**	2.30E-01	-0.05	0.1273	6.62E-01	9.38E-01
**GP5**	-0.1	0.1981	6.05E-01	9.02E-01	-0.05	0.1323	6.89E-01	9.47E-01	-0.19	0.2131	3.66E-01	8.16E-01	-0.09	0.1381	5.16E-01	8.83E-01
**GP6**	0.52	0.1699	**2.36E-03**	**2.26E-02**	0.17	0.1273	1.80E-01	4.36E-01	0.26	0.1776	1.33E-01	4.92E-01	-0.03	0.125	7.98E-01	9.49E-01
**GP7**	0.1	0.2049	6.24E-01	9.02E-01	-0.04	0.1328	7.47E-01	9.58E-01	0.05	0.2205	8.05E-01	9.49E-01	-0.05	0.1388	7.34E-01	9.49E-01
**GP8**	-0.17	0.2045	4.11E-01	7.93E-01	-0.05	0.1292	6.78E-01	9.47E-01	-0.12	0.2218	5.76E-01	9.11E-01	0.06	0.1323	6.51E-01	9.38E-01
**GP9**	-0.1	0.2035	6.21E-01	9.02E-01	-0.26	0.1313	**4.48E-02**	2.18E-01	0.03	0.2178	8.71E-01	9.87E-01	-0.19	0.1341	1.45E-01	4.92E-01
**GP10**	0.3	0.203	1.35E-01	3.99E-01	0.11	0.1325	4.00E-01	7.93E-01	-0.02	0.2106	9.28E-01	9.91E-01	0.01	0.1325	9.13E-01	9.91E-01
**GP11**	0.3	0.1981	1.29E-01	3.99E-01	0.01	0.1314	9.52E-01	9.80E-01	0.02	0.2079	9.41E-01	9.91E-01	-0.11	0.1341	4.10E-01	8.21E-01
**GP12**	0.03	0.192	8.78E-01	9.58E-01	-0.02	0.1311	8.88E-01	9.58E-01	0.05	0.2056	8.09E-01	9.49E-01	0.01	0.1366	9.17E-01	9.91E-01
**GP13**	-0.15	0.1909	4.20E-01	7.93E-01	-0.23	0.1301	6.99E-02	2.70E-01	-0.19	0.2052	3.36E-01	8.16E-01	-0.17	0.1328	1.95E-01	6.03E-01
**GP14**	-0.29	0.159	6.32E-02	2.70E-01	-0.17	0.122	1.66E-01	4.34E-01	-0.26	0.1717	1.27E-01	4.92E-01	-0.05	0.1239	6.90E-01	9.38E-01
**GP15**	-0.14	0.1897	4.40E-01	8.06E-01	-0.07	0.1303	5.77E-01	9.02E-01	-0.26	0.2019	1.91E-01	6.03E-01	-0.04	0.1322	7.34E-01	9.49E-01
**GP16**	-0.51	0.2008	**1.15E-02**	7.84E-02	0.05	0.133	6.97E-01	9.47E-01	-0.37	0.2162	8.09E-02	4.59E-01	0.11	0.1376	4.36E-01	8.47E-01
**GP17**	-0.35	0.1956	6.81E-02	2.70E-01	0.06	0.133	6.22E-01	9.02E-01	-0.25	0.2078	2.21E-01	6.54E-01	0.12	0.1386	3.72E-01	8.16E-01
**GP18**	-0.58	0.1659	**4.71E-04**	**6.40E-03**	-0.04	0.1273	7.30E-01	9.55E-01	-0.46	0.1769	**8.45E-03**	9.57E-02	0.09	0.1295	4.94E-01	8.83E-01
**GP19**	-0.59	0.1982	**2.66E-03**	**2.26E-02**	0.03	0.1325	8.22E-01	9.58E-01	-0.49	0.2112	**1.79E-02**	1.53E-01	0.12	0.1372	3.66E-01	8.16E-01
**GP20**	-0.35	0.1973	7.55E-02	2.70E-01	-0.1	0.1303	4.50E-01	8.06E-01	-0.33	0.21	1.09E-01	4.92E-01	0.06	0.1317	6.48E-01	9.38E-01
**GP21**	-0.5	0.2	**1.12E-02**	7.84E-02	0.09	0.1326	5.14E-01	8.96E-01	-0.49	0.2151	**2.17E-02**	1.64E-01	0.11	0.1379	4.01E-01	8.21E-01
**GP22**	-0.19	0.2041	3.45E-01	7.32E-01	0.16	0.1324	2.09E-01	4.91E-01	-0.14	0.2152	5.19E-01	8.83E-01	0.22	0.1377	1.13E-01	4.92E-01
**GP23**	-0.92	0.1852	**9.41E-07**	**6.40E-05**	-0.03	0.1298	8.40E-01	9.58E-01	-0.58	0.1892	**2.08E-03**	**2.91E-02**	0.13	0.127	3.06E-01	8.09E-01
**GP24**	-0.76	0.1964	**1.18E-04**	**2.00E-03**	0.32	0.1309	**1.49E-02**	9.20E-02	-0.64	0.2074	**1.79E-03**	**2.91E-02**	0.41	0.1353	**2.14E-03**	**2.91E-02**

*False discovery rate was controlled using Benjamini–Hochberg method; **Model 1 – adjustment for age; ***Model 2 – adjustment for age, BMI, diabetes, and smoking

Table S5. Statistical analysis of sex-stratified associations between 24 quantitative IgG glycosylation traits and coronary artery disease during the two-year follow up

	Model 1**								Model 2***							
	Women				Men				Women				Men			
Glycan peak	Effect	SE	p-value	p_{adj}-value*	Effect	SE	p-value	p_{adj}-value*	Effect	SE	p-value	p_{adj}-value*	Effect	SE	p-value	p_{adj}-value*
**GP1**	0.15	0.112	1.89E-01	6.76E-01	0	0.0879	9.60E-01	9.98E-01	0.16	0.1208	1.98E-01	5.85E-01	0.01	0.0917	9.17E-01	9.90E-01
**GP2**	-0.08	0.0867	3.59E-01	8.41E-01	-0.02	0.079	7.72E-01	9.41E-01	-0.1	0.0956	3.03E-01	7.62E-01	-0.01	0.0788	9.06E-01	9.90E-01
**GP3**	0.12	0.1492	4.26E-01	8.93E-01	-0.03	0.0956	7.29E-01	9.41E-01	0.05	0.1619	7.44E-01	9.90E-01	-0.06	0.1008	5.35E-01	9.33E-01
**GP4**	0.03	0.1017	7.50E-01	9.41E-01	-0.03	0.0638	5.92E-01	9.41E-01	0	0.1105	9.94E-01	9.94E-01	-0.05	0.0668	4.93E-01	9.06E-01
**GP5**	-0.21	0.1989	2.89E-01	8.41E-01	-0.2	0.1271	1.13E-01	6.36E-01	-0.31	0.2164	1.54E-01	5.52E-01	-0.24	0.1336	7.41E-02	5.04E-01
**GP6**	0.05	0.0839	5.12E-01	8.93E-01	0.09	0.053	8.65E-02	6.26E-01	0.02	0.0907	8.30E-01	9.90E-01	0.11	0.0561	5.33E-02	4.61E-01
**GP7**	-0.06	0.083	4.63E-01	8.93E-01	-0.02	0.0755	7.66E-01	9.41E-01	-0.07	0.0906	4.59E-01	8.74E-01	0	0.0785	9.94E-01	9.94E-01
**GP8**	-0.19	0.0902	**3.43E-02**	5.83E-01	-0.03	0.0835	7.26E-01	9.41E-01	-0.15	0.0989	1.21E-01	5.37E-01	-0.01	0.087	8.80E-01	9.90E-01
**GP9**	-0.08	0.102	4.57E-01	8.93E-01	-0.11	0.0704	1.32E-01	6.36E-01	-0.09	0.1106	4.40E-01	8.74E-01	-0.1	0.074	1.92E-01	5.85E-01
**GP10**	-0.05	0.1027	6.49E-01	9.41E-01	0.16	0.0608	**9.71E-03**	2.45E-01	-0.03	0.1135	7.96E-01	9.90E-01	0.19	0.0628	**2.58E-03**	8.79E-02
**GP11**	-0.05	0.094	5.77E-01	9.41E-01	0.05	0.054	3.47E-01	8.41E-01	-0.08	0.1034	4.63E-01	8.74E-01	0.08	0.0567	1.53E-01	5.52E-01
**GP12**	-0.01	0.1024	9.55E-01	9.98E-01	-0.03	0.0631	6.86E-01	9.41E-01	-0.04	0.1111	7.44E-01	9.90E-01	-0.02	0.0638	7.83E-01	9.90E-01
**GP13**	-0.12	0.1171	3.21E-01	8.41E-01	0.12	0.0744	9.63E-02	6.26E-01	-0.14	0.1252	2.55E-01	7.23E-01	0.16	0.0752	**3.51E-02**	4.61E-01
**GP14**	-0.03	0.0976	7.56E-01	9.41E-01	-0.01	0.0641	8.77E-01	9.62E-01	-0.01	0.1065	9.46E-01	9.90E-01	0.01	0.0667	8.69E-01	9.90E-01
**GP15**	-0.12	0.1168	3.19E-01	8.41E-01	0.11	0.0655	8.87E-02	6.26E-01	-0.1	0.1285	4.51E-01	8.74E-01	0.13	0.0692	5.42E-02	4.61E-01
**GP16**	0.12	0.0815	1.50E-01	6.36E-01	-0.06	0.0604	2.85E-01	8.41E-01	0.16	0.0887	7.34E-02	5.04E-01	-0.07	0.0634	3.00E-01	7.62E-01
**GP17**	-0.14	0.1477	3.53E-01	8.41E-01	-0.11	0.094	2.36E-01	7.69E-01	-0.17	0.1609	2.86E-01	7.62E-01	-0.13	0.0992	1.87E-01	5.85E-01
**GP18**	0	0.11	9.88E-01	9.98E-01	-0.05	0.063	4.16E-01	8.93E-01	0.06	0.1195	6.17E-01	9.54E-01	-0.04	0.0663	5.08E-01	9.09E-01
**GP19**	-0.08	0.1117	4.92E-01	8.93E-01	0.09	0.0629	1.45E-01	6.36E-01	0.02	0.1215	8.96E-01	9.90E-01	0.11	0.066	9.82E-02	5.37E-01
**GP20**	-0.37	0.2219	1.01E-01	6.26E-01	-0.08	0.141	5.95E-01	9.41E-01	-0.36	0.2393	1.40E-01	5.52E-01	-0.15	0.1504	3.37E-01	8.19E-01
**GP21**	-0.05	0.2564	8.36E-01	9.62E-01	-0.03	0.1448	8.52E-01	9.62E-01	0.07	0.2817	8.08E-01	9.90E-01	-0.02	0.1537	8.95E-01	9.90E-01
**GP22**	-0.32	0.1237	**1.08E-02**	2.45E-01	0.18	0.0938	6.27E-02	6.09E-01	-0.29	0.1338	**3.22E-02**	4.61E-01	0.15	0.0984	1.26E-01	5.37E-01
**GP23**	0.17	0.1251	1.84E-01	6.76E-01	-0.08	0.0666	2.37E-01	7.69E-01	0.22	0.1358	1.12E-01	5.37E-01	-0.11	0.0694	1.15E-01	5.37E-01
**GP24**	-0.03	0.1269	7.89E-01	9.42E-01	0.06	0.0794	4.23E-01	8.93E-01	0.12	0.1377	3.86E-01	8.74E-01	0.07	0.0828	3.83E-01	8.74E-01

*False discovery rate was controlled using Benjamini–Hochberg method; **Model 1 – adjustment for age; ***Model 2 – adjustment for age, BMI, diabetes, and smoking
